# Criteria for evaluation of disease extent by ^123^I-metaiodobenzylguanidine scans in neuroblastoma: a report for the International Neuroblastoma Risk Group (INRG) Task Force

**DOI:** 10.1038/sj.bjc.6605621

**Published:** 2010-04-27

**Authors:** K K Matthay, B Shulkin, R Ladenstein, J Michon, F Giammarile, V Lewington, A D J Pearson, S L Cohn

**Affiliations:** 1 Department of Pediatrics, University of California San Francisco School of Medicine, San Francisco, CA 94143–0106, USA; 2 St Jude Children's Research Hospital, Radiological Sciences, Memphis, TN 38105–3678, USA; 3 Department of Paediatric Oncology, St Anna Kinderspital, Vienna, Austria; 4 Department of Paediatrics, Institut Curie, Paris 75248, France; 5 Service de Médecine Nucléaire, CHLS, Hospices civils de Lyon and Faculté de Médecine, EA 3738, UCB Lyon 1, France; 6 Department of Nuclear Medicine and PET, The Royal Marsden NHS Foundation Trust, Sutton, Surrey SM2 5PT, UK; 7 Section of Paediatrics, Institute of Cancer Research and Royal Marsden Hospital, Surrey SM2 5PT, UK; 8 Department of Pediatrics, The University of Chicago, Chicago, IL 60637, USA

**Keywords:** neuroblastoma, mIBG, response criteria, International Neuroblastoma Risk Group (INRG), minimal disease

## Abstract

**Background:**

Neuroblastoma is an embryonic tumour of the sympathetic nervous system, metastatic in half of the patients at diagnosis, with a high preponderance of osteomedullary disease, making accurate evaluation of metastatic sites and response to therapy challenging. Metaiodobenzylguanidine (mIBG), taken into cells via the norepinephrine transporter, provides a sensitive and specific method of assessing tumour in both soft tissue and bone sites. The goal of this report was to develop consensus guidelines for the use of mIBG scans in staging, response assessment and surveillance in neuroblastoma.

**Methods:**

The International Neuroblastoma Risk Group (INRG) Task Force, including a multidisciplinary group in paediatric oncology of North and South America, Europe, Oceania and Asia, formed a subcommittee on metastatic disease evaluation, including expert nuclear medicine physicians and oncologists, who developed these guidelines based on their experience and the medical literature, with approval by the larger INRG Task Force.

**Results:**

Guidelines for patient preparation, radiotracer administration, techniques of scanning including timing, energy, specific views, and use of single photon emission computed tomography are included. Optimal timing of scans in relation to therapy and for surveillance is reviewed. Validated semi-quantitative scoring methods in current use are reviewed, with recommendations for use in prognosis and response evaluation.

**Conclusions:**

Metaiodobenzylguanidine scans are the most sensitive and specific method of staging and response evaluation in neuroblastoma, particularly when used with a semi-quantitative scoring method. Use of the optimal techniques for mIBG in staging and response, including a semi-quantitative score, is essential for evaluation of the efficacy of new therapy.

Neuroblastoma, the most common extra-cranial solid tumour in children, originates as a primary tumour of the sympathetic nervous system but metastasises commonly to bone and bone marrow, resulting in a poor prognosis. The frequency and the diffuse nature of the bone and bone marrow metastatic sites mandate a reliable, quantitative and consistent method of evaluation to assess response to therapy.

Metaiodobenzylguanidine (mIBG) is a guanethidine derivative and an analogue of norepinephrine and is therefore specifically taken up and stored in tumours derived from tissues of sympathetic nervous system origin, which express the norepinephrine transporter (Mairs *et al*, 1994; Boyd *et al*, 1999; McCluskey *et al*, 2005; Moroz *et al*, 2007). Owing to the high specificity and sensitivity in neuroblastoma, ^123^I-mIBG imaging has superseded the use of ^99m^Tc -technetium bone scans for the detection of skeletal metastases in the majority of children with neuroblastoma, which take up the tracer in >90% of cases, and has been recommended by the last international consensus conference as a standard element of staging and response evaluation (Hadley and Rabe, 1986; Lumbroso *et al*, 1988; Brodeur *et al*, 1993; Hero *et al*, 2001).

There is strong rationale for the use of ^123^I-mIBG scintigraphy in assessing response to therapy, as it provides a very sensitive marker of unsuspected skeletal and nodal disease and functional evidence of residual active tumour. With the widespread use of therapeutic ^131^I-mIBG as a targeted radiopharmaceutical treatment of neuroblastoma both in newly diagnosed and relapsed patients, diagnostic mIBG scans will determine the eligibility for this modality (Garaventa *et al*, 1999; Gaze *et al*, 2005; Matthay *et al*, 2006, 2007; de Kraker *et al*, 2008; DuBois and Matthay, 2008). Previous studies have indicated that a positive mIBG scan after induction chemotherapy or just before myeloablative therapy may be a prognostic marker for a high likelihood of relapse (Ladenstein *et al*, 1998; Perel *et al*, 1999; Schmidt *et al*, 2008). Several publications have reported the use of semi-quantitative scoring systems to increase the precision and inter-observer reliability of this test, with success in correlating the results of mIBG scans with early response and event-free survival in some, but not all, reports (Ady *et al*, 1995; Suc *et al*, 1996; Frappaz *et al*, 2000; Hero *et al*, 2001; Matthay *et al*, 2003b; Katzenstein *et al*, 2004). As treatment of stage 4 neuroblastoma continues to be a challenge, with a high rate of relapse in bone and bone marrow, it is essential to have a quantitative and reliable measure of response in bone metastases to test the activity of new therapies for this disease (Messina *et al*, 2006). The previous international consensus conference on neuroblastoma staging and response criteria recommended mIBG scans as part of the evaluation, but did not clearly define how to interpret a partial response by mIBG scan (Brodeur *et al*, 1993).

To facilitate comparisons of disease extent and response, one of the goals of the International Neuroblastoma Risk Group (INRG) Task Force, first convened in 2004, was to reach international consensus on the standard procedures for performing, interpreting and scoring mIBG scans. To facilitate comparison of clinical trials performed throughout the world, the William Guy Forbeck Research Foundation sponsored an international conference in September 2005 by inviting experts from the major cooperative groups in North America, Europe and Japan, as well as investigators from Australia and China. They were organised into four committees. The statistical, surgical and biological committee developed the INRG risk classification based on the statistical analysis of relevant prognostic factors, the INRG Staging System based on image-defined surgical risk factors, and procedures for the analysis of relevant biological markers (Ambros *et al*, 2009; Cohn *et al*, 2009; Monclair *et al*, 2009). The fourth committee was established to develop standard guidelines for detection and quantification of metastatic disease, including minimal disease in bone marrow and blood. One subcommittee reached a consensus on the techniques for semi-quantitative evaluation of bone marrow disease by immunocytology and RT–PCR (Beiske *et al*, 2009); the current subcommittee, composed of oncologists and nuclear medicine physicians, developed the guidelines for evaluation and standardised comparison of metastatic disease detected by ^123^I-mIBG scans, reported in this paper.

## Standardised procedures for mIBG scintigraphy

For details on the mIBG scan procedure, refer Shulkin and Shapiro (1998), Olivier *et al* (2003) and Bombardieri *et al* (2003).

### Thyroid blockade

Thyroid blockade is important to protect the organ from unnecessary irradiation from radioactive iodide that may dissociate from the mIBG. Thyroid blockade for ^123^I-mIBG scans can be achieved using aqueous iodine solution, oral potassium iodide (100 mg adult or 2 mg kg^–1^ children) or potassium iodate commencing 2–24 h before radiopharmaceutical injection and continuing for 1 day after, in accordance with local protocols or the European guidelines (Olivier *et al*, 2003). If a patient is allergic to iodine, oral potassium perchlorate may be substituted, given three times daily starting 2–24 h before and continuing for 2 days after, at a dose of 8 mg kg^–1^ (400 mg for adult).

### Drug interactions

Many classes of medicine have the potential to interfere with mIBG uptake and storage, so care should be taken before prescribing medications around the time of mIBG scan (Giammarile *et al*, 2008). The most commonly encountered agents that interfere with mIBG uptake and retention in children are alpha- and beta-adrenergic antagonists, such as pseudoephedrine and labetolol (Babich *et al*, 1997). Pseudoephedrine is a decongestant found in many over-the-counter cough and cold preparations, and labetolol is a beta-adrenergic antagonist for blood pressure control. The latter is usually quite effective for treatment of neuroblastoma-associated hypertension, but must be discontinued for several days before mIBG administration. Phenothiazines may interfere with mIBG uptake and should be avoided as sedatives before imaging. Phenylpropanolamine is another cough and cold preparation that also interferes with mIBG uptake and retention, but is no longer widely available. Serotonin re-uptake inhibitors have been listed as possibly inhibitory, but early laboratory data did not support their ability to inhibit mIBG uptake (Guilloteau *et al*, 1988), although some more recent reports show that venlafaxine and duloxetine can block norepinephrine reuptake (Lindauer *et al*, 2008; Tuccori *et al*, 2009). Cocaine, tricyclic antidepressants and reserpine are very rarely encountered inhibitors of mIBG uptake in children, but a tumour without mIBG uptake should always raise the question of possible interfering substances; however, it is rare that uptake is completely blocked.

### Radiopharmaceutical

Metaiodobenzylguanidine labelled with ^123^I is the radiopharmaceutical of choice in children for high-quality functional imaging of neuroblastoma. The gamma emission energy of 159 keV from ^123^I is more suitable for imaging than the 364 keV from ^131^I, and the differences in terms of radiation burden permit injection of 10- to 20-fold higher activities with ^123^I compared with ^131^I. The radiopharmaceutical is injected slowly over 0.5–1 min, usually via a peripheral vein, and flushed with saline. Rapid injection is contraindicated as it may cause adverse effects (vomiting, tachycardia, pallor abdominal pain). If possible, injection of central venous catheters should be avoided for technical reasons such as visualisation of the catheter. If a central venous catheter is used, it should be flushed well after injection and the site noted.

The sensitivity of detection with mIBG increases with increased activity injected, as shown for both ^131^I-mIBG and ^123^I-mIBG pre-therapy diagnostic scans compared with immediate post-treatment ^131^I-mIBG scans (Parisi *et al*, 1992; Hickeson *et al*, 2004). Furthermore, in the high-risk neuroblastoma patient group the radiation hazard is far less than the risk resulting from false-negative or false-positive scanning results (Stabin and Gelfand, 1998). Since longer scanning times may result in motion artefact, a sufficient count rate is necessary to obtain scintigrams of good quality within a short scanning time. Although rigorous paediatric dose-finding studies have not been performed, the European Association of Nuclear Medicine recommends that the administered activity should be calculated on a reference adult dose of 370 MBq scaled down for body weight (or body surface area) using the factors adjusted for weight in Table 1 of the Lassmann publication, with a minimum activity of 80 MBq (Jacobs *et al*, 2005; Lassmann *et al*, 2007). The need for high-quality images in patients with this often lethal malignancy and the danger of under-staging patients because of inadequate counts from the study, outweigh the theoretical risks from a slightly higher dose of the radiotracer (Bombardieri *et al*, 2003). Good hydration before and after the injection will lower the radiation burden and reduce bladder activity, which could interfere with evaluation of the pelvis. Radiation dose per unit activity administered for ^123^I-labelled mIBG is shown in Table 1, although in cases of pathological uptake it is more difficult to estimate the organ and whole body dose (Stabin and Gelfand, 1998).

### Image acquisition

Sedation is usually not required for a technically satisfactory examination, except for children between 1 and 3 years, and others who are unable to cooperate. Using ^123^I-mIBG, imaging is acquired between 20 and 24 h after the injection. Selected delayed images at 48 h may be useful in cases with equivocal findings, such as abdominal uptake that cannot be differentiated from the bowel (Rufini *et al*, 1996). Some institutions have chosen to obtain very early images, for example, at 4 h after injection, in addition to the routine 24-h images. Tumour uptake may become more distinct at later times compared with physiological uptake. In most institutions, early images are not obtained and not thought helpful.

As the principal energy of ^123^I is 159 keV, we recommend low-energy, high-resolution collimators, although scatter from additional low-abundance high-energy photon emission can degrade image quality. Therefore, it is possible to use a medium energy collimator and higher acquisition times, to minimise scatter with a similar sensitivity. The pixel size is approximately 2 mm, which requires a 256 × 256 matrix (preferred) or 128 × 128 with zoom.

### Views

The highest-quality images are obtained by having the child as close as possible to the camera face. Spot images of the entire body, including the skull (anterior, posterior and lateral views), chest (anterior and posterior views), abdomen (anterior and posterior views), pelvis (with empty bladder, anterior and posterior views), upper and lower limbs (anterior and posterior views) are the traditional method, but can be time consuming. However, whole body scan imaging with additional spot images including lateral views of the skull is often used and is equally acceptable. Spot views, if obtained, should be acquired in both the anterior and posterior planes from the top of the head through the pelvis. Anterior images of the lower extremities only are acceptable in institutions in which a dual-headed gamma camera is not available. Lateral images are sometimes valuable to discriminate between areas of overlapping uptake, for example, in the abdomen or pelvis, especially if the bladder is not empty.

Minimum 10 min per view for spot images (or 250 Kcounts for the skull and the trunk and 100 Kcounts for the lower limbs) is a suitable compromise between best image quality and limitation of scanning time. A fixed time of imaging is preferable to allow direct comparison of intensity between sets of images without image manipulation. For whole body scanning, a scan speed of 5 cm min^–1^ is appropriate when available. A whole body scan will require approximately 15 min in a 1-year-old, 17 min in a 2-year-old, 22 min in a 5-year-old, 28 min in a 10-year-old and 34 min in a 15-year-old.

### SPECT imaging

The feasibility of single photon emission computed tomography (SPECT) will depend on the child's cooperation and on the equipment available (multiple head camera). However, in cases in which uncertainty exists as to the exact site of mIBG activity, SPECT should be performed, even if sedation is required. The abdomen is the area in which this is most likely to occur, and less frequently, the chest. Lesions in or close to the liver, as well as close to the bladder or any other area of intense physiological uptake are particularly good indications to add SPECT, because some tumour sites can only be observed on SPECT (Gelfand *et al*, 1994; Rufini *et al*, 1995, 1996). Single photon emission computed tomography allows better comparison with anatomical imaging data, in particular when image fusion with CT or MR image is possible, or a combined SPECT–CT scanner is available (Tang *et al*, 2001; Rozovsky *et al*, 2008).

Single photon emission computed tomographyacquisition should be performed on a 128 × 128 matrix, 3° steps, with 30–35 s per step. Iterative reconstruction with a low-pass post-filter is preferred to filter back-projection reconstruction. If the acquisition time is too long and the child cannot or will not cooperate, SPECT using 6° instead of 3° steps or using a 64 × 64 matrix with shorter times per frame can be used.

## mIBG for staging at diagnosis

^123^I-mIBG scans are essential for initial staging of neuroblastoma, as they have a specificity of 85–96% (Leung *et al*, 1997). A recent study using blinded readers and concomitant pathology, CT scan and clinical information showed that ^123^I-mIBG scintigraphy had a sensitivity of 88% and a specificity of 83% (Vik *et al*, 2009). Occasionally, false-positive readings may occur because of uptake in mature ganglioneuroma or other neuroendocrine tumours, or because of physiological uptake that may be mistaken for tumour in the adrenal gland, salivary gland, nasopharynx, brown fat or excretion through renal pelvis and bladder (Pfluger *et al*, 2003). False-negative scans may be observed in approximately 10% of neuroblastomas that do not concentrate mIBG, owing to low expression of the norepinephrine transporter (Carlin *et al*, 2003), or owing to blood–brain barrier or large areas of scar or necrosis (Matthay *et al*, 2003a). In addition, very small amounts of bone marrow tumour will often not be detected, and therefore mIBG scan must be supplemented with bilateral bone marrow biopsy (Hero *et al*, 2001; Kushner *et al*, 2003). Metaiodobenzylguanidine score at diagnosis may be prognostic, as higher scores, indicating a high body burden of tumour, have been associated with a poorer outcome in some studies. For example, Suc *et al* (1996) have shown that an initial semi-quantitative score of >4 was independently associated with failure to achieve complete remission after induction chemotherapy, although most other studies showed only a trend or no significance to the initial mIBG score (Perel *et al*, 1999; Matthay *et al*, 2003b; Katzenstein *et al*, 2004; Yanik *et al*, 2010). Ongoing prospective studies of large numbers of uniformly treated patients in North America and Europe will further elucidate the prognostic significance of the initial mIBG score.

Single-photon emission computed tomography views increase the accuracy of geographical location of metastases, help differentiate from physiological areas of uptake and increase the sensitivity of detection on a lesion-by-lesion basis (Gelfand *et al*, 1994; Rufini *et al*, 1996). Single photon emission computed tomography views are generally not used in calculating the mIBG semi-quantitative score, because they are not routinely performed at all institutions or on a uniform protocol. In non-paediatric institutions, because of the longer time required for young children with additional sedation, and increased expense, they may not be regularly obtained. In the study by Vik *et al* (2009), SPECT views only marginally increased the sensitivity from 88 to 91%. Although additional information was gained in 65% of cases regarding the precise anatomic location of uptake, Curie scores would not have been substantially altered. However, the addition of SPECT views may be critical in cases in which there is a question regarding physiological uptake *vs* tumour uptake, or for precise localisation of a tumour focus that is critical for patient management (e.g., distinguishing a vertebral lesion from the adjacent pulmonary parenchyma). The addition of low-dose CT to SPECT (SPECT/CT) for both lesion localisation and attenuation correction has promise in providing more precise determination of the anatomic location of disease (Tang *et al*, 2001; Rozovsky *et al*, 2008).

### Other radiopharmaceuticals to assess disease

For those patients whose tumours are negative for mIBG uptake at diagnosis, the ^99m^Technetium MDP bone scan is the standard test recommended to evaluate skeletal metastases (Gordon *et al*, 1990; Brodeur *et al*, 1993; Perel *et al*, 1999). However, the low specificity of this test and the difficulty interpreting uptake in young children with actively growing bones make investigation of alternative methods preferable. Several exploratory reports of the use of ^18^F-deoxyglucose positron emission tomography (FDG-PET) scans suggest that this test also has a high specificity and sensitivity. However, as FDG-PET measures a metabolic characteristic of the tumour, while mIBG assesses the catecholamine reuptake pathway, false positives may be observed in inflammatory lesions, and there are also occasions when tumours may be FDG negative and mIBG positive, or vice versa (Shulkin *et al*, 1996; Kushner *et al*, 2001; Sharp *et al*, 2009). In newly diagnosed metastatic neuroblastoma, and in relapsed neuroblastoma, in which the most common sites of involvement are bone and bone marrow, recent studies suggest higher sensitivity for mIBG than FDG-PET, because of greater ability to detect the bone lesions, although the FDG-PET scans are often more sensitive for small soft tissue tumours and nodal metastases (Sharp *et al*, 2009; Taggart *et al*, 2009). Owing to the physiological brain uptake of FDG, it may be less sensitive than mIBG for lesions of the cranial vault (Kushner *et al*, 2001). The clinical value of FDG-PET in neuroblastoma patients is at present not well defined and the routine use of FDG-PET, especially in case of intention to use this as a substitute for ^123^I-mIBG scintigraphy, is not justified. In mIBG-negative tumours, the use of somatostatin receptor scintigraphy may be considered in selected individual patients, although uptake of ^111^In-pentetreotide is more common in favourable prognosis neuroblastoma (Schilling *et al*, 2000). Whole body MRI is also a sensitive test for neuroblastoma tumours, including bone and bone marrow metastases, although the specificity is much lower than mIBG (Pfluger *et al*, 2003). Further prospective and comparative studies will be needed to assess the relative merits of these methods.

## Timing of mIBG scans for response assessment and surveillance

Metaiodobenzylguanidine scans should be performed in all high-risk patients at diagnosis, before high-dose myeloablative therapy, and before and after therapy given for minimal residual disease. Other time points may be added according to assessments required to evaluate the particular treatment regimen and as specified for surveillance post-therapy. Metaiodobenzylguanidine scans have been shown to be the most sensitive method for detecting both residual bone disease and unsuspected asymptomatic relapse in high-risk neuroblastoma (Kushner *et al*, 2009; Taggart *et al*, 2009). In a study of 113 patients with asymptomatic relapse, ^123^I-mIBG had 82% sensitivity, compared with bone scan (36%) or bone marrow (34%) for detection of tumour. In comparison with ^18^FDG-PET scan, mIBG was significantly more sensitive for detection of bone lesions in relapsed neuroblastoma, with 94% sensitivity compared with 43% sensitivity in 122 individual bone lesions (Taggart *et al*, 2009). Intermediate- and low-risk patients should have mIBG scans at diagnosis, end of therapy and as surveillance at 6-month intervals until 1 year for low-risk stage 2 and 2 years after therapy for intermediate-risk patients. Although low- and intermediate-risk neuroblastoma have an excellent overall survival >90%, the event-free survival is only approximately 80% for stage 2 (Perez *et al*, 2000), stage 3 (Rubie *et al*, 1998), stage 4 and unfavourable biology 4 s (Nickerson *et al*, 2000; Schmidt *et al*, 2000; Hero *et al*, 2008; De Bernardi *et al*, 2009), suggesting that this modest surveillance schedule is reasonable to detect relapsing patients.

## Semi-quantitative mIBG scoring

### Historical development and assessment by the different scoring systems

To evaluate the prognostic effect of tumour burden at diagnosis and to be able to quantify response and define a partial response (50% reduction of all disease sites) by mIBG scan, semi-quantitative scoring systems have been developed (Ady *et al*, 1995; Suc *et al*, 1996; Perel *et al*, 1999; Frappaz *et al*, 2000; Matthay *et al*, 2003b; Katzenstein *et al*, 2004; Messina *et al*, 2006; Lewington *et al*, 2009). This improves the concordance between readers, and enables one to differentiate a simple improvement with the disappearance of a lesion, or decreased intensity of lesions that can be subjective, from a significant response that would qualify as PR. The scoring systems are all quite similar with minor variations, and take into account the frequent diffuse nature of skeletal involvement and the lack of precision if one attempted simply to count individual lesions.

**Figure 1:**
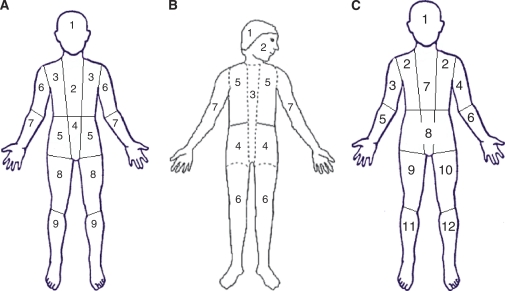
(**A**)^123^I-mIBG scoring method 1: method 1 divides the skeleton into nine segments to view osteomedullary involvement, and adds a tenth sector that counts any soft tissue involvement to the score. The extension score for method 1 is graded as: 0, no sites per segment; 1, one site per segment; 2, more than one site per segment; and 3, diffuse involvement (>50% of the segment). (**B**) ^123^I-mIBG scoring Frappaz-method 2: method 2 divides the skeleton into seven segments. The intensity score for method 2 is graded as: 0, no uptake; 1, doubtful uptake; 2, obvious but mild uptake; 3, strong uptake, with a maximum score of 21. Soft tissue involvement is noted separately from the score. (**C**) ^123^I-mIBG scoring SIOPEN-method 3: method 3 divides the skeleton into 12 anatomic segments. The extension score for method 3 is graded as: 0, no sites per segment, 1, one discrete site per segment; 2, two discrete lesions; 3, three discrete lesions; 4, >3 discrete foci or a single diffuse lesions involving <50% of a bone; 5, diffuse involvement of >50–95% whole bone; 6, diffuse involvement of the entire bone.

**Figure 2:**
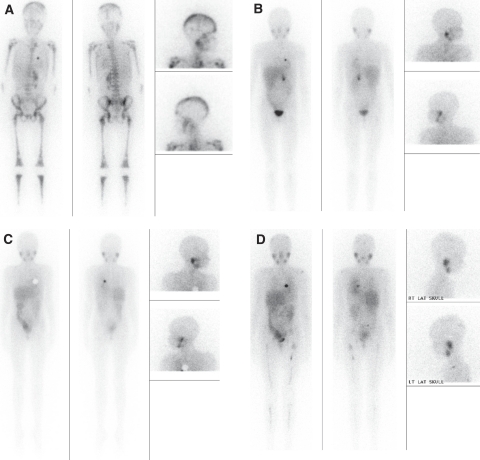
^123^I-mIBG scans and semi-quantitative scores on an 8 ½ -year-old female with widespread skeletal involvement of stage 4 neuroblastoma. Injection artefact from the portacath site in the left chest is observed in all images. The scores according to the three different methods most used are given; the Curie score (method 1) is currently the standard of comparison for other scoring variations. (**A**) At diagnosis: method 1 (Curie)= 26 (by segment 3, 3, 3, 3, 3, 3, 1, 3, 3, 1); method 2 (Frappaz)= 21 (by segment: 3, 3, 3, 3, 3, 3, 3); method 3 (SIOPEN)= 61 (by segment: 5, 5, 5, 5, 4, 4, 6, 6, 6, 6, 4, 5). (**B**) During induction: method 1= 1 for the soft tissue; method 2= 0; method 3= 0. (**C**) End therapy. Note the disappearance of soft tissue involvement because of surgical resection of primary tumour: method 1= 0; method 2= 0; method 3= 0. (**D**) At relapse: method 1= 13 (by segment: 2, 1, 1, 1, 1, 1, 0, 3, 3, 0); method 2=9 (by segment: 2, 0, 2, 1, 1, 2, 1); method 3=29 (by segment: 3, 1, 1, 1, 0, 0, 1, 4, 5, 5, 4, 4).

The most common scoring methods divide the skeleton into anatomical sectors, then give each sector an individual score for extension (quantity of metastases) and intensity (strength of uptake). The sum of the scores for each sector gives a separate mIBG score for extent and for intensity. Examples are shown in Figures 1 and 2.

The first method reported was developed at the Curie Institute in France (method 1; ‘Curie’) (Ady *et al*, 1995). It divides the skeleton into nine segments to view osteomedullary involvement, and adds a tenth sector that counts any soft tissue involvement (Figure 1A). The extension score for method 1 is graded as: 0, no sites per segment; 1, one site per segment; 2, more than one site per segment; and 3, diffuse involvement (>50% of the segment). The intensity score is graded as: 0, for no uptake; 1, for doubtful uptake; 2, for definite uptake less than liver; and 3, for intense uptake greater than that of liver. Thus, the maximum score for either extension or intensity would be 30. The Curie score (method 1) has been shown to have a good inter-observer concordance of 92 and 95% in two independent studies (Matthay *et al*, 2003b; Messina *et al*, 2006). It has been validated in France and is now widely used in North America for the New Approaches to Neuroblastoma Therapy consortium and the Children's Oncology Group (COG). The Curie score has been shown in both retrospective and prospective analyses of scores at the end of induction chemotherapy to correlate well with overall response and with event-free survival (Matthay *et al*, 2003b; Messina *et al*, 2006; Yanik *et al*, 2010). It was used as a reliable method for evaluating response in patients with relapsed disease undergoing new therapies (Matthay *et al*, 2006, 2009; Messina *et al*, 2006).

In 1996, Suc *et al* published a modification of the Curie score in which the skeleton was divided into seven segments, and reported that patients with a score >4 at diagnosis had a poorer outcome. This system was subsequently shown to have good inter-observer concordance, but did not show prognostic value for outcome on further study (Frappaz *et al*, 2000). The Frappaz score (method 2) uses seven segments of the skeleton, and initially did not include soft tissue in the score, but noted it separately (Figure 1B). Thus, for evaluation of skeletal disease, the methods were somewhat comparable, but for assessing overall response by mIBG, method 2 was less accurate as initially reported, as soft tissue metastases were not counted in the score, but noted separately (Messina *et al*, 2006). The score for method 2 was called an intensity score, and graded for each segment as: 0, no uptake; 1, doubtful uptake; 2, obvious but mild uptake; 3, strong uptake, with a maximum score of 21. A separate qualitative ‘diffusion’ score was entered as 0 if there was no uptake, 1 if uptake was spotty and 2 if uptake was diffuse, with a maximum score of 14 (Frappaz *et al*, 2000).

Perel *et al* (1999) published another minor variation on the Curie score, in which the skeleton was divided into 10 rather than 9 zones (the skull was divided into the calvarium and the base of the skull, as in the Frappaz method), and soft tissue involvement was ignored. Otherwise, this was identical to the Curie score. In a subsequent publication, this method was shown to have prognostic significance at the end of induction therapy, with a better outcome for patients having a score of <3 (Katzenstein *et al*, 2004).

A third major variation was recently presented by Lewington *et al* (2009) in a large group of neuroblastoma patients treated in the high-risk neuroblastoma SIOPEN study (method 3; ‘SIOPEN’). In the SIOPEN score, currently under prospective evaluation in Europe, the skeletal distribution of mIBG was recorded in 12 anatomical body segments as follows: skull, thoracic cage, proximal right upper limb, distal right upper limb, proximal left upper limb, distal left upper limb, spine, pelvis, proximal right lower limb, distal right lower limb, proximal left lower limb and distal left lower limb (Figure 1C). The extent and pattern of skeletal mIBG involvement was scored using a 0–6 scale to discriminate between focal discrete lesions and patterns of more diffuse infiltration. Each segment is scored as 0, no involvement; 1, one discrete lesion; 2, two discrete lesions; 3, three discrete lesions; 4, >3 discrete foci or a single diffuse lesion involving <50% of a bone; 5, diffuse involvement of >50 to 95% whole bone; 6, diffuse involvement of the entire bone, with a maximum score of 72. This method showed 95% concordance in a blinded review by six nuclear medicine physicians. It also proved slightly superior to the Frappaz score as a measure of response evaluation (Lewington *et al*, 2009).

### Response evaluation by mIBG score

For response evaluation, the relative extension scores are calculated by dividing the absolute post-therapy score by the absolute pre-therapy score. A relative score of 0.5 is considered a partial response; a relative score of 0 is a complete response (Matthay *et al*, 2003b). Alternatively, an absolute score can be shown to be a cut-off for a ‘good’ response (Ladenstein *et al*, 1998; Katzenstein *et al*, 2004). A recent study of mIBG scores in the COG study A3973 for 274 high-risk stage 4 neuroblastoma patients showed a significantly worse EFS for patients with scores >5 at the end of induction (Yanik *et al*, 2010). It must be always considered that any assessment of a prognostic factor is dependent on uniform therapy and the particular therapy given; therefore, results from one study may not be applicable to another with different therapy.

In general, the sum for the intensity score and that for the extension score correlate closely and do not change the conclusion (Matthay *et al*, 2003b). However, as the intensity score is slightly more subjective because of technical factors, showed lower concordance among readers, and did not change the response evaluation in two studies, it is not being used in recent and ongoing prospective studies (Matthay *et al*, 2003b; Messina *et al*, 2006; Yanik *et al*, 2010).

The Curie score was more reliable than the Frappaz score for evaluation of response in a blinded comparison, partly because of the fact that the Frappaz score did not incorporate soft tissue metastases into the score (Messina *et al*, 2006). The Curie extension score is currently being used in large prospective phase 3 COG trials for high-risk neuroblastoma in North America (A3973; ANBL0532) (Yanik *et al*, 2010). The SIOPEN score is the current method being used in Europe for the prospective phase 3 neuroblastoma trial. Although both methods have shown good inter-observer concordance and good correlation with outcome, at the current time, the Curie score has been tested more widely and over many years, and is less complex.

## Conclusion

We suggest these guidelines for image acquisition and analysis to facilitate high-quality studies in patients with neuroblastoma and to achieve a high degree of consistency in interpretation. A semi-quantitative method of image analysis is essential, and should show low inter-observer variability and high reproducibility, while providing a range of values for comparison with other indicators of disease extent and response. We have adopted these guidelines for application in upcoming cooperative neuroblastoma clinical trials to validate them and test their utility in the management of patients with neuroblastoma.

## Figures and Tables

**Table 1:** Radiation absorbed dose with ^123^I-mIBG scan (ICRP, 1998)

**Age**	**MBq administered**	***μ*Sv MBq^−1^**	**Effective dose per administration (mSv)**
Adult	370	13	4.81
15	340	17	5.79
10	240	26	6.25
5	170	37	6.30
1	100	68	6.79

## References

[bib1] Ady N, Zucker JM, Asselain B, Edeline V, Bonnin F, Michon J, Gongora R, Manil L (1995) A new 123I-MIBG whole body scan scoring method--application to the prediction of the response of metastases to induction chemotherapy in stage IV neuroblastoma. Eur J Cancer 31A: 256–261 7718334 10.1016/0959-8049(94)00509-4

[bib2] Ambros PF, Ambros IM, Brodeur GM, Haber M, Khan J, Nakagawara A, Schleiermacher G, Speleman F, Spitz R, London WB, Cohn SL, Pearson AD, Maris JM (2009) International consensus for neuroblastoma molecular diagnostics: report from the International Neuroblastoma Risk Group (INRG) Biology Committee. Br J Cancer 100: 1471–1482 19401703 10.1038/sj.bjc.6605014 PMC2694415

[bib3] Babich JW, Graham W, Fischman AJ (1997) Effect of adrenergic receptor ligands on metaiodobenzylguanidine uptake and storage in neuroblastoma cells. Eur J Nucl Med 24: 538–543 9142735 10.1007/BF01267686

[bib4] Beiske K, Burchill SA, Cheung IY, Hiyama E, Seeger RC, Chon SL, Pearson ADJ, Matthay KK (2009) Consensus criteria for sensitive detection of minimal neuroblastoma cells in bone marrow, blood and stem cell preparations by immunocytology and QRT-PCR: recommendations by the International Neuroblastoma Risk Group (INRG) Task Force. J Clin Oncol 100(10): 1627–1637 10.1038/sj.bjc.6605029 PMC2696761 19401690

[bib5] Bombardieri E, Aktolun C, Baum RP, Bishof-Delaloye A, Buscombe J, Chatal JF, Maffioli L, Moncayo R, Mortelmans L, Reske SN (2003) 131I/123I-metaiodobenzylguanidine (MIBG) scintigraphy: procedure guidelines for tumour imaging. Eur J Nucl Med Mol Imaging 30: BP132–BP139 14989227 10.1007/s00259-003-1357-0

[bib6] Boyd M, Cunningham SH, Brown MM, Mairs RJ, Wheldon TE (1999) Noradrenaline transporter gene transfer for radiation cell kill by 131I meta-iodobenzylguanidine. Gene Ther 6: 1147–1152 10455418 10.1038/sj.gt.3300905

[bib7] Brodeur GM, Pritchard J, Berthold F, Carlsen NL, Castel V, Castelberry RP, De Bernardi B, Evans AE, Favrot M, Hedborg F, Kaneko M, Lampert F, Lee REJ, Look AT, Pearson ADJ, Phillip T, Roald B, Sawada T, Seeger RC, Tsuchida Y, Voute PA (1993) Revisions of the international criteria for neuroblastoma diagnosis, staging, and response to treatment [see comments]. J Clin Oncol 11: 1466–1477 8336186 10.1200/JCO.1993.11.8.1466

[bib8] Carlin S, Mairs RJ, McCluskey AG, Tweddle DA, Sprigg A, Estlin C, Board J, George RE, Ellershaw C, Pearson AD, Lunec J, Montaldo PG, Ponzoni M, van Eck-Smit BL, Hoefnagel CA, van den Brug MD, Tytgat GA, Caron HN (2003) Development of a real-time polymerase chain reaction assay for prediction of the uptake of meta-[(131)I]iodobenzylguanidine by neuroblastoma tumors. Clin Cancer Res 9: 3338–3344 12960120

[bib9] Cohn SL, Pearson AD, London WB, Monclair T, Ambros PF, Brodeur GM, Faldum A, Hero B, Iehara T, Machin D, Mosseri V, Simon T, Garaventa A, Castel V, Matthay KK (2009) The International Neuroblastoma Risk Group (INRG) classification system: an INRG Task Force report. J Clin Oncol 27: 289–297 19047291 10.1200/JCO.2008.16.6785 PMC2650388

[bib10] De Bernardi B, Gerrard M, Boni L, Rubie H, Canete A, Di Cataldo A, Castel V, Forjaz de Lacerda A, Ladenstein R, Ruud E, Brichard B, Couturier J, Ellershaw C, Munzer C, Bruzzi P, Michon J, Pearson AD (2009) Excellent outcome with reduced treatment for infants with disseminated neuroblastoma without MYCN gene amplification. J Clin Oncol 27: 1034–1040 19171711 10.1200/JCO.2008.17.5877

[bib11] de Kraker J, Hoefnagel KA, Verschuur AC, van Eck B, van Santen HM, Caron HN (2008) Iodine-131-metaiodobenzylguanidine as initial induction therapy in stage 4 neuroblastoma patients over 1 year of age. Eur J Cancer 44(4): 551–556 18267358 10.1016/j.ejca.2008.01.010

[bib12] DuBois SG, Matthay KK (2008) Radiolabeled metaiodobenzylguanidine for the treatment of neuroblastoma. Nucl Med Biol 35(Suppl 1): S35–S48 18707633 10.1016/j.nucmedbio.2008.05.002 PMC2633223

[bib13] Frappaz D, Bonneu A, Chauvot P, Edeline V, Giammarile F, Siles S, Wioland M, Gomez F (2000) Metaiodobenzylguanidine assessment of metastatic neuroblastoma: observer dependency and chemosensitivity evaluation. The SFOP Group. Med Pediatr Oncol 34: 237–241 10742058 10.1002/(sici)1096-911x(200004)34:4<237::aid-mpo1>3.0.co;2-j

[bib14] Garaventa A, Bellagamba O, Lo Piccolo MS, Milanaccio C, Lanino E, Bertolazzi L, Villavecchia GP, Cabria M, Scopinaro G, Claudiani F, De Bernardi B (1999) 131I-metaiodobenzylguanidine (131I-MIBG) therapy for residual neuroblastoma: a mono-institutional experience with 43 patients. Br J Cancer 81: 1378–1384 10604736 10.1038/sj.bjc.6694223 PMC2362971

[bib15] Gaze MN, Chang YC, Flux GD, Mairs RJ, Saran FH, Meller ST (2005) Feasibility of dosimetry-based high-dose 131I-meta-iodobenzylguanidine with topotecan as a radiosensitizer in children with metastatic neuroblastoma. Cancer Biother Radiopharm 20: 195–199 15869455 10.1089/cbr.2005.20.195

[bib16] Gelfand MJ, Elgazzar AH, Kriss VM, Masters PR, Golsch GJ (1994) Iodine-123-MIBG SPECT versus planar imaging in children with neural crest tumors. J Nucl Med 35: 1753–1757 7965151

[bib17] Giammarile F, Chiti A, Lassmann M, Brans B, Flux G (2008) EANM procedure guidelines for 131I-meta-iodobenzylguanidine (131I-mIBG) therapy. Eur J Nucl Med Mol Imaging 35: 1039–1047 18274745 10.1007/s00259-008-0715-3

[bib18] Gordon I, Peters AM, Gutman A, Morony S, Dicks-Mireaux C, Pritchard J (1990) Skeletal assessment in neuroblastoma--the pitfalls of iodine-123-MIBG scans [see comments]. J Nucl Med 31: 129–134 2313350

[bib19] Guilloteau D, Chalon S, Baulieu JL, Huguet F, Desplanches G, Chambon C, Vilar MP, Pourcelot L, Besnard JC (1988) Comparison of MIBG and monoamines uptake mechanisms: pharmacological animal and blood platelets studies. Eur J Nucl Med 14: 341–344 3141186 10.1007/BF00254380

[bib20] Hadley GP, Rabe E (1986) Scanning with iodine-131 MIBG in children with solid tumors: an initial appraisal. J Nucl Med 27: 620–626 3712077

[bib21] Hero B, Hunneman DH, Gahr M, Berthold F (2001) Evaluation of catecholamine metabolites, mIBG scan, and bone marrow cytology as response markers in stage 4 neuroblastoma. Med Pediatr Oncol 36: 220–223 11464889 10.1002/1096-911X(20010101)36:1<220::AID-MPO1053>3.0.CO;2-6

[bib22] Hero B, Simon T, Spitz R, Ernestus K, Gnekow AK, Scheel-Walter HG, Schwabe D, Schilling FH, Benz-Bohm G, Berthold F (2008) Localized infant neuroblastomas often show spontaneous regression: results of the prospective trials NB95-S and NB97. J Clin Oncol 26: 1504–1510 18349403 10.1200/JCO.2007.12.3349

[bib23] Hickeson MP, Charron M, Maris JM, Brophy P, Kang TI, Zhuang H, Khan J, Nevrotski T (2004) Biodistribution of post-therapeutic versus diagnostic (131)I-MIBG scans in children with neuroblastoma. Pediatr Blood Cancer 42: 268–274 14752865 10.1002/pbc.10454

[bib24] ICRP (1998) Radiation dose to patients from radiopharmaceuticals (addendum 2 to ICRP publication 53). Ann ICRP 28: 1–126 10.1016/s0146-6453(99)00006-8 10840563

[bib25] Jacobs F, Thierens H, Piepsz A, Bacher K, Van de Wiele C, Ham H, Dierckx RA (2005) Optimised tracer-dependent dosage cards to obtain weight-independent effective doses. Eur J Nucl Med Mol Imaging 32: 581–588 15619101 10.1007/s00259-004-1708-5

[bib26] Katzenstein HM, Cohn SL, Shore RM, Bardo DM, Haut PR, Olszewski M, Schmoldt J, Liu D, Rademaker AW, Kletzel M (2004) Scintigraphic response by 123I-metaiodobenzylguanidine scan correlates with event-free survival in high-risk neuroblastoma. J Clin Oncol 22: 3909–3915 15459212 10.1200/JCO.2004.07.144

[bib27] Kushner BH, Kramer K, Modak S, Cheung NK (2009) Sensitivity of surveillance studies for detecting asymptomatic and unsuspected relapse of high-risk neuroblastoma. J Clin Oncol 27: 1041–1046 19171710 10.1200/JCO.2008.17.6107 PMC2667809

[bib28] Kushner BH, Yeh SD, Kramer K, Larson SM, Cheung NK (2003) Impact of metaiodobenzylguanidine scintigraphy on assessing response of high-risk neuroblastoma to dose-intensive induction chemotherapy. J Clin Oncol 21: 1082–1086 12637474 10.1200/JCO.2003.07.142

[bib29] Kushner BH, Yeung HW, Larson SM, Kramer K, Cheung NK (2001) Extending positron emission tomography scan utility to high-risk neuroblastoma: fluorine-18 fluorodeoxyglucose positron emission tomography as sole imaging modality in follow-up of patients. J Clin Oncol 19: 3397–3405 11454888 10.1200/JCO.2001.19.14.3397

[bib30] Ladenstein R, Philip T, Lasset C, Hartmann O, Garaventa A, Pinkerton R, Michon J, Pritchard J, Klingebiel T, Kremens B, Pearson A, Coze C, Paolucci P, Frappaz D, Gadner H, Chauvin F (1998) Multivariate analysis of risk factors in stage 4 neuroblastoma patients over the age of one year treated with megatherapy and stem-cell transplantation: a report from the European Bone Marrow Transplantation Solid Tumor Registry. J Clin Oncol 16: 953–965 9508178 10.1200/JCO.1998.16.3.953

[bib31] Lassmann M, Biassoni L, Monsieurs M, Franzius C, Jacobs F (2007) The new EANM paediatric dosage card. Eur J Nucl Med Mol Imaging 34: 796–798 17406866 10.1007/s00259-007-0370-0

[bib32] Leung A, Shapiro B, Hattner R, Kim E, de Kraker J, Ghazzar N, Hartmann O, Hoefnagel CA, Jamadar DA, Kloos R, Lizotte P, Lumbroso J, Rufini V, Shulkin BL, Sisson JC, Thein A, Troncone L (1997) Specificity of radioiodinated MIBG for neural crest tumors in childhood. J Nucl Med 38: 1352–1357 9293786

[bib33] Lewington V, Bar Sever Z, Lynch T, Giammarile F, McEwan A, Shulkin B, Staudenherz A, Ladenstein R (2009) Development of a new, semi-quantitative I-123 mIBG reporting method in high risk neuroblastoma. Eur J Nucl Med Mol Imaging 36: 334 27663238 10.1007/s00259-016-3516-0 PMC5214990

[bib34] Lindauer A, Siepmann T, Oertel R, Jung A, Ziemssen T, Jaehde U, Kirch W, Siepmann M (2008) Pharmacokinetic/pharmacodynamic modelling of venlafaxine: pupillary light reflex as a test system for noradrenergic effects. Clin Pharmacokinet 47: 721–731 18840027 10.2165/00003088-200847110-00003

[bib35] Lumbroso JD, Guermazi F, Hartmann O, Coornaert S, Rabarison Y, Leclere JG, Couanet D, Bayle C, Caillaud JM, Lemerle J et al (1988) Meta-iodobenzylguanidine (mIBG) scans in neuroblastoma: sensitivity and specificity, a review of 115 scans. Prog Clin Biol Res 271: 689–705 3261424

[bib36] Mairs RJ, Livingstone A, Gaze MN, Wheldon TE, Barrett A (1994) Prediction of accumulation of 131I-labelled meta-iodobenzylguanidine in neuroblastoma cell lines by means of reverse transcription and polymerase chain reaction. Br J Cancer 70: 97–101 7517173 10.1038/bjc.1994.256 PMC2033329

[bib37] Matthay KK, Brisse H, Couanet D, Couturier J, Benard J, Mosseri V, Edeline V, Lumbroso J, Valteau-Couanet D, Michon J (2003a) Central nervous system metastases in neuroblastoma: radiologic, clinical, and biologic features in 23 patients. Cancer 98: 155–165 12833468 10.1002/cncr.11448

[bib38] Matthay KK, Edeline V, Lumbroso J, Tanguy ML, Asselain B, Zucker JM, Valteau-Couanet D, Hartmann O, Michon J (2003b) Correlation of early metastatic response by 123I-metaiodobenzylguanidine scintigraphy with overall response and event-free survival in stage IV neuroblastoma. J Clin Oncol 21: 2486–2491 12829667 10.1200/JCO.2003.09.122

[bib39] Matthay KK, Quach A, Huberty J, Franc BL, Hawkins RA, Jackson H, Groshen S, Shusterman S, Yanik G, Veatch J, Brophy P, Villablanca JG, Maris JM (2009) Iodine-131--metaiodobenzylguanidine double infusion with autologous stem-cell rescue for neuroblastoma: a new approaches to neuroblastoma therapy phase I study. J Clin Oncol 27: 1020–1025 19171714 10.1200/JCO.2007.15.7628 PMC2738616

[bib40] Matthay KK, Tan JC, Villablanca JG, Yanik GA, Veatch J, Franc B, Twomey E, Horn B, Reynolds CP, Groshen S, Seeger RC, Maris JM (2006) Phase I dose escalation of iodine-131-metaiodobenzylguanidine with myeloablative chemotherapy and autologous stem-cell transplantation in refractory neuroblastoma: a new approaches to Neuroblastoma Therapy Consortium Study. J Clin Oncol 24: 500–506 16421427 10.1200/JCO.2005.03.6400

[bib41] Matthay KK, Yanik G, Messina J, Quach A, Huberty J, Cheng SC, Veatch J, Goldsby R, Brophy P, Kersun LS, Hawkins RA, Maris JM (2007) Phase II study on the effect of disease sites, age, and prior therapy on response to iodine-131-metaiodobenzylguanidine therapy in refractory neuroblastoma. J Clin Oncol 25: 1054–1060 17369569 10.1200/JCO.2006.09.3484

[bib42] McCluskey AG, Boyd M, Ross SC, Cosimo E, Clark AM, Angerson WJ, Gaze MN, Mairs RJ (2005) [131I]meta-iodobenzylguanidine and topotecan combination treatment of tumors expressing the noradrenaline transporter. Clin Cancer Res 11: 7929–7937 16278418 10.1158/1078-0432.CCR-05-0982

[bib43] Messina JA, Cheng SC, Franc BL, Charron M, Shulkin B, To B, Maris JM, Yanik G, Hawkins RA, Matthay KK (2006) Evaluation of semi-quantitative scoring system for metaiodobenzylguanidine (mIBG) scans in patients with relapsed neuroblastoma. Pediatr Blood Cancer 47: 865–874 16444675 10.1002/pbc.20777

[bib44] Monclair T, Brodeur GM, Ambros PF, Brisse HJ, Cecchetto G, Holmes K, Kaneko M, London WB, Matthay KK, Nuchtern JG, von Schweinitz D, Simon T, Cohn SL, Pearson AD (2009) The International Neuroblastoma Risk Group (INRG) staging system: an INRG Task Force report. J Clin Oncol 27: 298–303 19047290 10.1200/JCO.2008.16.6876 PMC2650389

[bib45] Moroz MA, Serganova I, Zanzonico P, Ageyeva L, Beresten T, Dyomina E, Burnazi E, Finn RD, Doubrovin M, Blasberg RG (2007) Imaging hNET reporter gene expression with 124I-MIBG. J Nucl Med 48: 827–836 17475971 10.2967/jnumed.106.037812

[bib46] Nickerson HJ, Matthay KK, Seeger RC, Brodeur GM, Shimada H, Perez C, Atkinson JB, Selch M, Gerbing RB, Stram DO, Lukens J (2000) Favorable biology and outcome of stage IV-S neuroblastoma with supportive care or minimal therapy: a Children's Cancer Group study. J Clin Oncol 18: 477–486 10653863 10.1200/JCO.2000.18.3.477

[bib47] Olivier P, Colarinha P, Fettich J, Fischer S, Frokier J, Giammarile F, Gordon I, Hahn K, Kabasakal L, Mann M, Mitjavila M, Piepsz A, Porn U, Sixt R, van Velzen J (2003) Guidelines for radioiodinated MIBG scintigraphy in children. Eur J Nucl Med Mol Imaging 30: B45–B50 12658506 10.1007/s00259-003-1138-9

[bib48] Parisi MT, Matthay KK, Huberty JP, Hattner RS (1992) Neuroblastoma: dose-related sensitivity of MIBG scanning in detection. Radiology 184: 463–467 1620849 10.1148/radiology.184.2.1620849

[bib49] Perel Y, Conway J, Kletzel M, Goldman J, Weiss S, Feyler A, Cohn SL (1999) Clinical impact and prognostic value of metaiodobenzylguanidine imaging in children with metastatic neuroblastoma. J Pediatr Hematol Oncol 21: 13–18 10029806 10.1097/00043426-199901000-00004

[bib50] Perez CA, Matthay KK, Atkinson JB, Seeger RC, Shimada H, Haase GM, Stram DO, Gerbing RB, Lukens JN (2000) Biologic variables in the outcome of stages I and II neuroblastoma treated with surgery as primary therapy: a children's cancer group study. J Clin Oncol 18: 18–26 10623689 10.1200/JCO.2000.18.1.18

[bib51] Pfluger T, Schmied C, Porn U, Leinsinger G, Vollmar C, Dresel S, Schmid I, Hahn K (2003) Integrated imaging using MRI and 123I metaiodobenzylguanidine scintigraphy to improve sensitivity and specificity in the diagnosis of pediatric neuroblastoma. AJR Am J Roentgenol 181: 1115–1124 14500242 10.2214/ajr.181.4.1811115

[bib52] Rozovsky K, Koplewitz BZ, Krausz Y, Revel-Vilk S, Weintraub M, Chisin R, Klein M (2008) Added value of SPECT/CT for correlation of MIBG scintigraphy and diagnostic CT in neuroblastoma and pheochromocytoma. AJR Am J Roentgenol 190: 1085–1090 18356459 10.2214/AJR.07.2107

[bib53] Rubie H, Michon J, Plantaz D, Peyroulet MC, Coze C, Frappaz D, Chastagner P, Baranzelli MC, Maechinaud F, Boutard P, Lutz P, Perel Y, Leverger G, de Lumley L, Millot F, Staephan JL, Margueritte G, Hartmann O (1998) Unresectable localized neuroblastoma: improved survival after primary chemotherapy including carboplatin-etoposide. Neuroblastoma Study Group of the Sociaetae Franecaise d’Oncologie Paediatrique SFOP. Br J Cancer 77: 2310–2317 9649151 10.1038/bjc.1998.384 PMC2150389

[bib54] Rufini V, Fisher GA, Shulkin BL, Sisson JC, Shapiro B (1996) Iodine-123-MIBG imaging of neuroblastoma: utility of SPECT and delayed imaging. J Nucl Med 37: 1464–1468 8790194

[bib55] Rufini V, Giordano A, Di Giuda D, Petrone A, Deb G, De Sio L, Donfrancesco A, Troncone L (1995) [123I]MIBG scintigraphy in neuroblastoma: a comparison between planar and SPECT imaging. Q J Nucl Med 39: 25–28 9002745

[bib56] Schilling FH, Bihl H, Jacobsson H, Ambros PF, Martinsson T, Borgstrom P, Schwarz K, Ambros IM, Treuner J, Kogner P (2000) Combined (111)In-pentetreotide scintigraphy and (123)I-mIBG scintigraphy in neuroblastoma provides prognostic information. Med Pediatr Oncol 35: 688–691 11107147 10.1002/1096-911x(20001201)35:6<688::aid-mpo44>3.0.co;2-7

[bib57] Schmidt M, Simon T, Hero B, Schicha H, Berthold F (2008) The prognostic impact of functional imaging with (123)I-mIBG in patients with stage 4 neuroblastoma >1 year of age on a high-risk treatment protocol: results of the German Neuroblastoma Trial NB97. Eur J Cancer 44: 1552–1558 18424129 10.1016/j.ejca.2008.03.013

[bib58] Schmidt ML, Lukens JN, Seeger RC, Brodeur GM, Shimada H, Gerbing RB, Stram DO, Perez C, Haase GM, Matthay KK (2000) Biologic factors determine prognosis in infants with stage IV neuroblastoma: a Prospective Children's Cancer Group Study. J Clin Oncol 18: 1260–1268 10715296 10.1200/JCO.2000.18.6.1260

[bib59] Sharp SE, Shulkin BL, Gelfand MJ, Salisbury S, Furman WL (2009) 123I-MIBG scintigraphy and 18F-FDG PET in neuroblastoma. J Nucl Med 50: 1237–1243 19617326 10.2967/jnumed.108.060467

[bib60] Shulkin BL, Hutchinson RJ, Castle VP, Yanik GA, Shapiro B, Sisson JC (1996) Neuroblastoma: positron emission tomography with 2-[fluorine-18]-fluoro-2-deoxy-D-glucose compared with metaiodobenzylguanidine scintigraphy. Radiology 199: 743–750 8637999 10.1148/radiology.199.3.8637999

[bib61] Shulkin BL, Shapiro B (1998) Current concepts on the diagnostic use of MIBG in children. J Nucl Med 39: 679–688 9544682

[bib62] Stabin MG, Gelfand MJ (1998) Dosimetry of pediatric nuclear medicine procedures. Q J Nucl Med 42: 93–112 9695662

[bib63] Suc A, Lumbroso J, Rubie H, Hattchouel JM, Boneu A, Rodary C, Robert A, Hartmann O (1996) Metastatic neuroblastoma in children older than one year: prognostic significance of the initial metaiodobenzylguanidine scan and proposal for a scoring system. Cancer 77: 805–811 8616776 10.1002/(sici)1097-0142(19960215)77:4<805::aid-cncr29>3.0.co;2-3

[bib64] Taggart D, Han MM, Quach A, Groshen S, Ye W, Villablanca JG, Jackson HA, Aparici CM, Darlson D, Maris J, Hawkins R, Matthay KK (2009) Comparison of 123I-metaiodobenzylguanidine (MIBG) scan and 18F-fluorodeoxyglucose positron emission tomography (FDG-PET) to evaluate response after 131I-MIBG therapy for neuroblastoma. J Clin Oncol 27(32): 5343–5349 19805691 10.1200/JCO.2008.20.5732 PMC2773221

[bib65] Tang HR, Da Silva AJ, Matthay KK, Price DC, Huberty JP, Hawkins RA, Hasegawa BH (2001) Neuroblastoma imaging using a combined CT scanner-scintillation camera and 131I-MIBG. J Nucl Med 42: 237–247 11216522

[bib66] Tuccori M, Testi A, Antonioli L, Fornai M, Montagnani S, Ghisu N, Colucci R, Corona T, Blandizzi C, Del Tacca M (2009) Safety concerns associated with the use of serotonin reuptake inhibitors and other serotonergic/noradrenergic antidepressants during pregnancy: a review. Clin Ther 31(Part 1): 1426–1453 19698902 10.1016/j.clinthera.2009.07.009

[bib67] Vik TA, Pfluger T, Kadota R, Castel V, Tulchinsky M, Farto JC, Heiba S, Serafini A, Tumeh S, Khutoryansky N, Jacobson AF (2009) (123)I-mIBG scintigraphy in patients with known or suspected neuroblastoma: results from a prospective multicenter trial. Pediatr Blood Cancer 52: 784–790 19185008 10.1002/pbc.21932

[bib68] Yanik GA, Parisi MT, Naranjo A, Matthay KK, London WB, Mcgrady PW, Kreissman SG, Shulkin BL (2010) MIBG scoring as a prognostic indicator in patients with stage 4 neuroblastoma. a COG study. J Clin Oncol 2010. Supplement ASCO Proceedings, in press

